# A Knowledge, Attitude, and Practice Survey on Medication Safety in Korean Older Adults: An Analysis of an Ageing Society

**DOI:** 10.3390/healthcare9101365

**Published:** 2021-10-14

**Authors:** Mijin Lee, Kyungim Kim, Kiyon Rhew, Kyung-Hee Choi

**Affiliations:** 1 College of Pharmacy, Sunchon National University, 255 Jungang-ro, Suncheon 57922, Korea; mjlee0844@gmail.com; 2 Institute of Pharmaceutical Science, Korea University, 2511 Sejong-ro, Sejong 30019, Korea; kim_ki@korea.ac.kr; 3 College of Pharmacy, Korea University, 2511 Sejong-ro, Sejong 30019, Korea; 4 College of Pharmacy, Dongduk Women’s University, 60 Hwarang-ro 13-gil, Seongbuk-gu, Seoul 02748, Korea; kiyon@dongduk.ac.kr

**Keywords:** aged, health knowledge, attitude, and practice, over-the-counter drugs, health education, self-medication

## Abstract

Background: Older adults have certain limitations in acquiring and understanding information regarding medication safety. This study surveyed their medication habits and analysed the importance of relevant education to improve knowledge, attitudes, and practice (KAP). Methods: Our survey included adults aged 65 years or older. We developed a questionnaire on medication safety based on the KAP model. To identify the interrelationships among KAP, we calculated the correlation coefficients using Pearson’s correlation analysis. A t-test was performed to verify the differences in KAP associated with the respondents’ medication safety education experience. Results: We found that 79.4% of respondents self-administered their medications. Of the respondents, 28.2% had received medication safety education. Overall, the respondents had typical levels of knowledge, attitude responses, and behavioural practices associated with medication safety. The results showed significant differences between knowledge and practice; those who were educated on medication safety performed higher levels of safe practice than those who were not (*p* < 0.05). Conclusion: The KAP survey confirmed that knowledge about the safe use of medication positively affected older adults’ attitudes and practices. To improve their medication usage habits, older adults should receive well-organised medication safety education.

## 1. Introduction

The ageing of the global population is a widespread phenomenon, and currently, it seems to be happening faster than in the past. The World Health Organization estimates that the proportion of individuals older than 65 years in 2000 is expected to more than double by 2050 [1]. The number of older individuals and older couples living alone is also growing because of rapid changes in social structures. Moreover, the number of medications that older adults take by themselves has also increased. Households comprising only older adults require more socioeconomic attention to improve their healthcare as well as financial status for better quality of life [2,3].

Ageing is accompanied by several physical and mental issues not experienced by younger individuals, such as a decline of physical and mental capabilities, including worsening sight and hearing, increased physical frailty, and difficulty adapting to rapid social changes. They also experience changes in their pharmacokinetic and pharmacodynamic characteristics [4,5]. Furthermore, the prevalence of chronic diseases increases with age. More than half of older adults have three or more chronic diseases; therefore, older adults attend clinics often and take prescribed medications. They also tend to use non-prescribed or over-the-counter (OTC) medications to relieve various symptoms without first obtaining advice from a clinician [6,7]. Because of the increase in the older population and their chronic conditions, older adults have become the largest consumers of prescription and OTC drugs [8]. They are more vulnerable to adverse drug effects and are exposed to the risks associated with using multiple or inappropriate medications [4,9,10]. Taking many types of medications can cause confusion, thereby leading to serious health consequences or even fatalities due to improper use [11].

Self-medication refers to individuals recognising their own symptoms, making a self-diagnosis, and choosing and using medications by themselves [12,13]. Self-medication with OTC drugs can be an economical treatment choice; furthermore, OTC drugs are legally accessible at pharmacies [14,15]. However, this creates concerns because older adults are more likely to misuse medications and may have difficulties in understanding the adverse effects associated with the medications they choose to manage their symptoms with [12,14].

Older individuals comprise a vulnerable group with limitations in acquiring and understanding drug safety information [16]. For example, they often have difficulty accessing information through the use of mobile devices, computers, and the Internet; moreover, they often have vision and/or hearing problems or mental factors that act as barriers to the acquisition of information [17,18,19,20]. Therefore, safe medication use should be emphasised and needs to be investigated in this group of individuals.

We surveyed older individuals to investigate their medication habits and to identify their unmet needs associated with safe medication use. The knowledge, attitude, and practice (KAP) model was used to develop a questionnaire for these individuals. The original KAP survey has been widely used to obtain health behaviour information [21,22,23]. Although knowledge is related to attitude and practice, it does not perfectly predict behaviours [24,25]. Therefore, it was necessary to determine how interactions among these factors affect human behaviours. Knowledge represents more than having information; rather, it refers to the ability to collect, maintain, and use information [26]. Existing studies have shown that knowledge can be gained through education and experience and that attitudes—including the concepts of perceptions, feelings, beliefs, and behavioural tendencies—can be changed through health education. Practice refers to applying the acquired knowledge to actions [26,27,28].

Based on these attributes, we adopted the KAP model to analyse knowledge gaps, cultural beliefs, and behavioural patterns among older adults [29]. This study surveyed experiences, education, and knowledge regarding safe medication use among older adults and analysed how medication safety education can affect their KAP.

## 2. Materials and Methods

### 2.1. Study Design and Data Collection

This was a cross-sectional study designed to examine older adults’ drug use and to evaluate the relationships among their KAP and medication safety habits.

We enrolled adults aged 65 years or older who were able to participate in social activities and excluded those who were unable to live independently, who could not ambulate without assistance, and those with severe cognitive impairments. The survey included older adults living in various regions, such as the capital city, urban areas, and rural regions of Korea to ensure a data sample with various social backgrounds. To recruit participants, we visited senior community welfare centres and retail pharmacies and conveyed the purpose and scope of this study. The questionnaire was distributed in paper form, considering that older adults are generally not familiar with social networking tools. All participants provided written informed consent forms, and we collected the data from May to June 2017. All procedures were approved by the Institutional Review Board of Korea University (KU-IRB-17-58-P-1). This study was conducted in accordance with the ethical guidelines stipulated in the Declaration of Helsinki.

### 2.2. Survey Design

The survey had two parts and comprised a total of 44 questions. The first part collected the participants’ demographic data, their experiences with medication safety education provided by senior organisations or local healthcare centres, their satisfaction with education, and their actual OTC drug use habits. The second part investigated the effects of knowledge on their attitudes and practices associated with medication safety. Furthermore, the second part included 25 questions: 10 were knowledge-based; eight were attitude-based, and seven were practice-based. The medication safety education programme referred to in the questionnaire was created by the Korean Pharmaceutical Association and is provided to senior organisations and local healthcare centres [30].

Using the KAP model as a foundation for our questionnaire, we aimed to identify the relationships among KAP and medication use by older individuals. Except for the questions on demographic characteristics, the respondents answered questions using a 5-point Likert scale ranging from “strongly disagree” to “strongly agree”, with a higher total score indicating higher levels of KAP associated with medication use.

Four investigators validated and revised the questionnaire. Cronbach’s α test was conducted to assess the internal consistency of the questionnaire, with the following results: knowledge, 0.607; attitude, 0.759; and practice, 0.612. A Cronbach’s α coefficient of 0.6 or more indicates an acceptable internal consistency [31].

### 2.3. Statistics Analysis

We only analysed the responses to the questionnaires when more than half of the questions were answered and excluded questionnaires from the analysis when the respondents did not include information about their age or sex.

Categorical variables such as sex, residential area, age group, residence type, and method of taking medication are presented as frequencies and percentages. Self-medication habits and experiences with medication safety education are also represented as frequencies and percentages. Continuous variables are presented as means and standard deviations (SD). Pearson’s correlation coefficient was used to identify the interrelationships between knowledge and attitude, knowledge and practice, and attitude and practice. The R-value of each item was interpreted as a moderate correlation of 0.4 or more, which was generally considered to be significant [32]. A t-test was performed to verify the differences in KAP based on educational experience.

Statistical significance was set at *p* ≤ 0.05 and determined by bilateral test using IBM SPSS Statistics software (version 25.0; IBM, Armonk, NY, USA).

## 3. Results

Of the 150 respondents, the data of 131 older adults were included in the analysis. Of the 19 excluded respondents, 6 were younger than 65 years, 7 submitted questionnaires with missing responses on age or sex, and 6 submitted questionnaires that were incomplete as more than half of the questions had missing responses.

### 3.1. General Characteristics of the Respondents

The demographic characteristics of the respondents are shown in Table 1. Of the 131 respondents analysed, 22.9% were male. Regarding their place of residence, 47.3%, 26.0%, and 26.7% lived in the capital city, an urban area, and a rural area, respectively. The largest age group comprised individuals aged 65–70 years (45.0%). The most common residence status was living alone or living with spouse. We found that 79.4% of the respondents took their medications by themselves.

### 3.2. Self-Medication Status and Medication Safety Use Education

**Figure 1:**
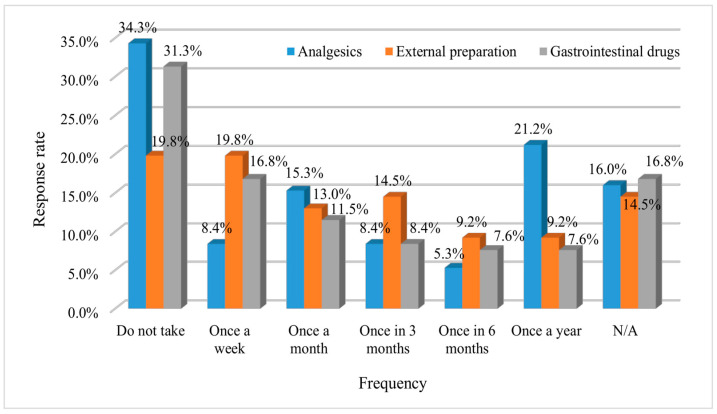
Average self-medication over 1 year. N/A = no answer. The use of analgesics is shown in blue. External preparation (orange) = patches or ointments. Gastrointestinal drugs (grey) = anti-diarrhoeal, antacid, digestant, or laxative.

Among the 113 respondents (excluding non-responders), 49.6% reported self-administration of analgesics within the past year, while 32 respondents reported using analgesics within the past month. External OTC drugs such as patches and ointments, and gastrointestinal drugs, such as anti-diarrhoeal, antacids, digestants, and laxatives, were most frequently used; with most respondents reporting to have used them within the past week (Figure 1).

**Figure 2:**
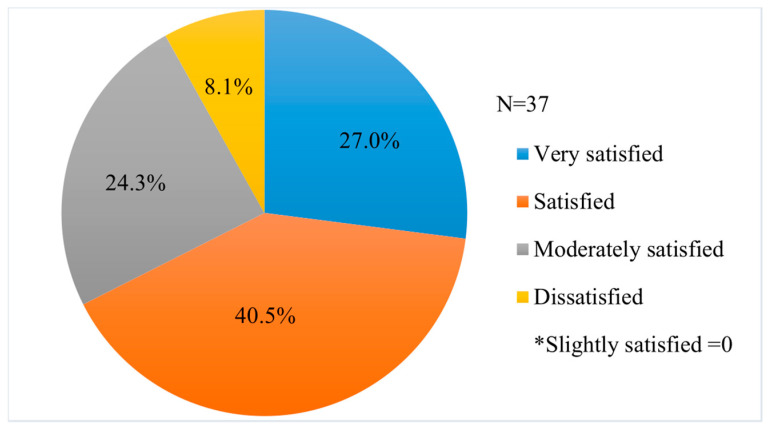
Satisfaction with medication safety education.

Table 2 shows the results of the medication safety educational experience of the respondents and their satisfaction with that experience (Figure 2). Of all respondents, 28.2% had medication safety educational experience, and only three respondents reported that they were dissatisfied with that experience. Most respondents (78 respondents; 59.54%) reported that they preferred on-site lectures on safe drug use, regardless of their previous educational experience, while 28 (21.27%) respondents indicated that they preferred audio–visual education (Table 3).

Questions and answers related to KAP are presented in Table 4. The results show that overall, the levels of KAP associated with medication safety were average. Older adults used good practice while taking medication (4.56 ± 0.98); however, these individuals had a low perception of adverse drug reactions (2.56 ± 1.23). The results of Pearson’s correlation analysis confirmed correlations among KAP associated with medication safety. Knowledge showed a significantly positive correlation with both attitude (r = 0.454; *p* < 0.05) and practice (r = 0.440; *p* < 0.05). Attitude and practice (r = 0.448; *p* < 0.05) also had a significant positive correlation (Table 5).

We also aimed to determine how drug safety use education affects attitudes, behaviours, and knowledge. Student’s t-test was conducted to determine whether there was a significant difference among KAP based on whether the individuals had previously received medication safety education. Knowledge (t = −2.340; *p* < 0.05) and practice (t = −3.143; *p* < 0.05) associated with education experience were significantly different. A comparison of the average KAP scores indicated that those who had received safe drug use education had higher scores (3.67 ± 0.51) than those who had not (3.40 ± 0.54; t = −0.758; *p* < 0.05; Table 6).

## 4. Discussion

In the current ageing society, older adults form a large part of the worldwide population. As they start to live independently as “old people,” their quality of life is influenced by new major issues [33].

In this study, we surveyed independently living older adults to investigate their medication habits and the factors that affect their safety when using medications. The characteristics of the individuals enrolled in this study were similar to those enrolled in the study by Kharicha, who assessed the health risks of older adults in the United Kingdom and found that 37% of the population 65 years and older lived alone [34]. We found that most older adults took their medications by themselves and that self-medication without assistance from others decreased their treatment adherence. This result is comparable to that reported in a study by Park et al., which was conducted on older adults with chronic diseases who were living alone [35].

These results imply that older adults can become isolated from information about medication. The physical and mental health levels of older adults living alone were poorer than those of older adults living with their families [35,36,37]. One study also reported that many ageing individuals were using medication that had expired because they had difficulty reading the expiry date on the package label [38].

Current society is rapidly changing, and older adults tend to have difficulty in coping with changing environments, often due to health problems affecting their physical and cognitive functions. They are less adaptable to new trends in acquiring information and more vulnerable to various diseases and discomforts. In other words, ageing is a factor that can prevent sufficient access to media and updated information. Therefore, although proper drug use is crucial for the wellbeing of elderly individuals with chronic diseases or geriatric-related pain, they often lack knowledge about medication because of their difficulties in obtaining information.

Individuals can obtain OTC drugs because they are more accessible than prescription drugs and can be purchased in large quantities at one time; therefore, these medications are often stored for long periods. During this study, we selected analgesics, external usage formulations such as patches and ointments, and gastrointestinal drugs because they are the most frequently consumed OTC products in Korea [39]. We showed that these OTC drugs are used often by older adults, which is consistent with the findings of the study by Amoako on older adults and their self-medication practices [13]. Furthermore, most respondents reported the habitual use of these OTC drugs more than once per week or more than once per month. There are some concerns about the health management and OTC drug use practices of older adults because some of them indicated that their medications include nonsteroidal anti-inflammatory drugs or H2 blockers listed in the Beers criteria [40,41,42,43]. The Beers criteria, which were initially developed by an expert consensus panel in 1991, are the most widely used criteria for determining medications that are inappropriate for use by older individuals due to their high risk of adverse drug events [41]. Furthermore, because of their propensity to use OTC drugs arbitrarily, older adults need to have accurate knowledge of their medications to avoid serious risks.

This study aimed to investigate the awareness of safe medication use in older adults using a questionnaire developed based on the KAP model and to confirm the correlations among each element of KAP. In this model, K represents knowledge of diseases and medications, A represents the attitude toward those diseases and medications, and P represents practice or preventive actions to protect against diseases and unsafe medication use [27]. The results confirmed that knowledge related to the safe use of medications positively affected attitudes and practice, which was in line with the results of the study by Hope that investigated the associations among adherence, knowledge, and skills of patients with congestive heart failure and confirmed that the medication use behaviours of the subjects improved after they received proper education [44]. Several studies have revealed that older adults often lack knowledge about side effects, usage methods, and dosage of their medication; however, most individuals are aware of the purpose of their medication [45,46,47]. Additionally, Tesfamariam et al. reported that poor knowledge was one factor that resulted in inappropriate OTC medication use [47]. These results emphasise the importance of and the need for medication safety education, especially among older adults using OTC drugs. Furthermore, Kim et al. reported the impact of education on preventing medical errors [48]. These findings have great implications for the importance of drug safety education. Therefore, it is necessary to provide this education and to reinforce its adherence for it to become effective.

In this study, although the survey participants had a little safe drug use education experience, they reported high satisfaction with that education and indicated that they prefer education in the form of on-site lectures and audio–visual materials rather than other methods such as textbooks. Some studies also found that education using audio–visual methods is more effective than that using brochures or pamphlets [49,50,51]. Therefore, these preferred types of educational materials need to be developed and adapted to help ensure the safety of these individuals.

Few questionnaires have used the KAP model for medication safety practiced by older adults. Therefore, this study is valuable for two reasons: First, the survey was conducted by directly contacting older adults in various regions. Second, this study revealed actual OTC medicine use habits and the importance of knowledge among older adults. Most importantly, we confirmed that older adults also recognise their need for education and are able to identify their preferred form of education.

This study was limited by its use of a paper survey rather than one available through the use of newer tools such as multimedia. However, because the questionnaire was paper and administered face-to-face to the respondents, it may be more suitable for older adults who find technology to be challenging.

## 5. Conclusions

The quality of life of the ageing population is an emerging issue. Older adults are living independently more often, but their capabilities are declining. The correlations among safe medication use and KAP of older adults found during this study underscore the need for systemic medication safety education for older adults. Therefore, it is necessary to design and implement various educational programmes that can be easily accessed by older adults so as to improve their awareness of practices related to safe medication use.

## Figures and Tables

**Table 1:** Demographic characteristics (*n* = 131).

Characteristics	Participants (*n*)	Education Experience (*n*, %)
Sex		
Male	30	12 (40.0)
Female	101	25 (24.7)
Residence area		
Capital city	62	16 (25.8)
Urban	34	15 (44.1)
Rural	35	6 (17.1)
Age, years		
65–70	59	17 (28.8)
70–75	29	5 (17.2)
75–80	15	4 (26.7)
80–85	22	9 (40.9)
85–90	6	2 (33.3)
Residence type		
Alone or with spouse	81	24 (29.6)
With descendants	39	10 (25.6)
Other ^1^	5	1 (20.0)
No answer	6	2 (33.3)
Medication use		
Self-administered	104	33 (31.7)
Administered by spouse/descendants (family)	9	1 (11.1)
Other	2	1 (50.0)
No answer	16	2 (12.5)

^1^ With a health care worker or in a nursing home.

**Table 2:** Experience receiving education about safe medication use.

Experience	Total (*n* = 131)	
	*n*	%
Yes	37	28.2
No	82	62.6
Do not remember	2	1.5
No answer	10	23.8

**Table 3:** Preferences regarding safe medication education (*n* = 131).

Classification	Education Experience, *n* (%)			
	Yes	No	Do Not Remember	No Answer
On-site lecture	23 (17.6)	48 (36.6)	2 (1.5)	5 (3.8)
Audio–visual education	7 (5.3)	20 (15.3)	-	1 (0.8)
Field study	2 (1.5)	1 (0.8)	-	-
Learning via home visit from a health care professional	2 (1.5)	2 (1.5)	-	1 (0.8)
Do not know	-	1 (0.8)	-	-
No answer	3 (2.3)	10 (7.6)	-	3 (2.3)
Total	37 (28.2)	82 (62.6)	2 (1.5)	10 (7.6)

**Table 4:** Assessment of knowledge, attitude, and practice associated with medication safety (*n* = 131).

KAP	Questions	Mean Score (SD)
K ^1^	The medication should be administered with water.	4.56 (0.98)
	The medication should be administered according to the prescribed dosage and usage.	4.28 (1.48)
	It is okay to change the shape of the medication, such as breaking the tablet in half.	3.32 (1.55)
	If you are older, there are some medications you should not use.	3.38 (1.34)
	You will get better sooner with more types of medication.	3.81 (1.38)
	It is recommended that drugs should be stored in the refrigerator.	2.92 (1.48)
	You know the effects and side effects of drugs that you buy or are prescribed.	2.56 (1.23)
	When reading the medication guide, you fully understand the contents.	3.03 (1.31)
	When receiving medication counselling, you fully understand the content.	3.55 (1.25)
	You know both the dosage and the benefits of your household medication.	3.37 (1.32)
A ^2^	You can buy as many medications as you want at any time via phone or the Internet.	3.67 (1.45)
	If you have unused prescription medication remaining, you can use it whenever you have similar symptoms.	3.40 (1.46)
	If it is good for my body, then it does not matter what medication I use.	3.89 (1.42)
	Dietary supplements have no side effects.	3.41 (1.48)
	Drugs advertised on television and radio are reliable.	3.45 (1.32)
	Medication has no expiration date.	4.00 (1.36)
	Remaining medications or expired medications can be thrown away in the household trash or sewer.	3.70 (1.50)
	If you use the medication for a long time, you might experience side effects.	3.53 (1.33)
P ^3^	You have good knowledge of the safe use of medications.	2.95 (1.25)
	You ask the pharmacist in detail about the medication you have bought.	3.39 (1.33)
	You buy medications based on the experiences of family and friends.	3.98 (1.27)
	When you experience any unusual symptoms after using the medication, you suspect those symptoms might be side effects.	3.11 (1.32)
	If you suspect side effects of medications, you consult your doctor or pharmacist.	3.65 (1.32)
	You stop using the medication as soon as the symptoms of the illness are no longer present.	2.77 (1.40)
	You keep the medication with its explanatory insert.	3.33 (1.58)

SD = standard deviation; KAP = knowledge, attitude, and practice. ^1^ Knowledge. ^2^ Attitude. ^3^ Practice.

**Table 5:** Correlations among knowledge, attitude, and practice associated with medication safety (*n* = 131).

Correlation	K ^1^	A ^2^	P ^3^
K ^1^	1	-	-
A ^2^	0.454 *	1	-
P ^3^	0.440 *	0.448 *	1

* *p* < 0.05. ^1^ Knowledge. ^2^ Attitude. ^3^ Practice.

**Table 6:** Comparison of knowledge, attitude, and practice scores associated with education experience (*n* = 131).

KAP	Educational Experience	n	Mean	SD	t	*p*
K ^1^	No	82	3.41	0.54	−2.340 *	0.021
	Yes	44	3.66	0.58		
A ^2^	No	82	3.59	0.83	−1.231	0.221
	Yes	44	3.78	0.82		
P ^3^	No	82	3.20	0.65	−3.143 *	0.002
	Yes	44	3.57	0.60		
KAP total	No	82	3.40	0.54	−2.758 *	0.007
	Yes	44	3.67	0.51		

* *p* < 0.05. KAP = knowledge, attitude, and practice. ^1^ Knowledge. ^2^ Attitudes. ^3^ Practice.

## Data Availability

The data presented in this study are available upon request from the corresponding author.

## References

[1] World Health Organization (2008). Men, Ageing and Health: Achieving Health Across the Life Span. https://www.who.int/ageing/publications/men/en/.

[2] Abd Wahab M.S. (2015). The relevance of educating doctors, pharmacists and older patients about potentially inappropriate medications. Int. J. Clin. Pharm..

[3] Piekut M. (2020). Living standards in one-person households of the elderly population. Sustainability.

[4] Rockwood K., Mitnitski A., Song X., Steen B., Skoog I. (2006). Long-term risks of death and institutionalization of elderly people in relation to deficit accumulation at age 70. J. Am. Geriatr. Soc..

[5] American Geriatrics Society Expert Panel on the Care of Older Adults with Multimorbidity (2012). Guiding principles for the care of older adults with multimorbidity: An approach for clinicians. J. Am. Geriatr. Soc..

[6] Kim L.D., Koncilja K., Nielsen C. (2018). Medication management in older adults. Clevel. Clin. J. Med..

[7] Kinsey J.D., Nykamp D. (2017). Dangers of nonprescription medicines: Educating and counseling older adults. Consult. Pharm..

[8] Francis S.-A., Barnett N., Denham M. (2005). Switching of prescription drugs to over-the-counter status. Drugs Aging.

[9] Singh S., Bajorek B. (2014). Defining “elderly” in clinical practice guidelines for pharmacotherapy. Pharm. Pr..

[10] Hines L.E., Murphy J.E. (2011). Potentially harmful drug–drug interactions in the elderly: A review. Am. J. Geriatr. Pharmacother..

[11] Ruiz M.E. (2010). Risks of self-medication practices. Curr. Drug Saf..

[12] World Health Organization (2000). Guidelines for the Regulatory Assessment of Medicinal Products for Use in Self-Medication. https://apps.who.int/iris/handle/10665/66154.

[13] Amoako E.P., Richardson-Campbell L., Kennedy-Malone L. (2003). Self-medication with over-the-counter drugs among elderly adults. J. Gerontol. Nurs..

[14] Zaprutko T., Koligat D., Michalak M., Wieczorek M., Józiak M., Ratajczak P., Szydłowska K., Miazek J., Kus K., Nowakowska E. (2016). Misuse of OTC drugs in Poland. Health Policy.

[15] Delavar F., Poor S.P., Negarandeh R. (2020). The effects of self-management education tailored to health literacy on medication adherence and blood pressure control among elderly people with primary hypertension: A randomized controlled trial. Patient Educ. Couns..

[16] Dillon C., Taragano F.E. (2016). Activity and lifestyle factors in the elderly: Their relationship with degenerative diseases and depression. AIMS Med. Sci..

[17] Brayne C., Gill C., Huppert F.A., Barkley C., Gehlhaar E., Girling D.M., O’Connor D.W., Paykel E.S. (1995). Incidence of clinically diagnosed subtypes of dementia in an elderly population. Br. J. Psychiatry.

[18] Pernambuco C.S., Rodrigues B.M., Bezerra J.C.P., Carrielo A., Fernandes A.D.D.O., Vale R.G.D.S., Dantas E.H.M. (2012). Quality of life, elderly and physical activity. Health.

[19] Olaya B., Moneta M.V., Bobak M., Haro J.M., Demakakos P. (2019). Cardiovascular risk factors and memory decline in middle-aged and older adults: The English longitudinal study of ageing. BMC Geriatr..

[20] Launiala A. (2009). How much can a KAP survey tell us about people’s knowledge, attitudes and practices? Some observations from medical anthropology research on malaria in pregnancy in Malawi. Anthr. Matters.

[21] Lakhan R., Sharma M. (2010). A study of knowledge, attitudes and practices (KAP) survey of families toward their children with intellectual disability in Barwani. India. Asia Pac. Disabil. Rehabil. J..

[22] Klett-Tammen C.J., Krause G., Seefeld L., Ott J.J. (2015). Determinants of tetanus, pneumococcal and influenza vaccination in the elderly: A representative cross-sectional study on knowledge, attitude and practice (KAP). BMC Public Health.

[23] Diwan V.K., Sachs L., Wahlström R. (1997). Practice-knowledge-attitudes-practice: An explorative study of information in primary care. Soc. Sci. Med..

[24] Nichols S.D.C. (1974). Nutrition Knowledge, Attitudes and Practices of Public Health Nurses in Greater Vancouver. Master’s Thesis.

[25] Badran I.G. (1995). Knowledge, attitude and practice the three pillars of excellence and wisdom: A place in the medical profession. East. Mediterr. Health. J..

[26] Wan T.T., Rav-Marathe K., Marathe S. (2016). A systematic review of KAP-O framework for diabetes. Med Res. Arch..

[27] Ajzen I., Fishbein M. (1980). Understanding Attitudes and Predicting Social Behavior.

[28] World Health Organization (2014). Knowledge, Attitudes, and Practices (KAP) Surveys During Cholera Vaccination Campaigns: Guidance for Oral Cholera Vaccine Stockpile Campaigns. Working Group on Monitoring and Evaluation.

[29] Breiner H., Ford M., Gadsden V.L. (2016). Parenting knowledge, attitudes, and practices. Parenting Matters: Supporting Parents of Children Ages 0–8.

[30] Korean Pharmaceutical Association. http://www.paadu.or.kr/sub/safety_introduce.asp?menu_current=8.

[31] Nunnally J.C. (1994). Psychometric Theory.

[32] Schober P., Boer C., Schwarte L.A. (2018). Correlation coefficients: Appropriate use and interpretation. Anesth. Analg..

[33] Doležalová J., Tóthová V., Neugebauer J., Sadílek P. (2021). Impact of Selected geriatric syndromes on the quality of life in the population aged 60 and older. Healthcare.

[34] Kharicha K., Iliffe S., Harari D., Swift C., Gillmann G., Stuck A.E. (2007). Health risk appraisal in older people 1: Are older people living alone an ‘at-risk’ group?. Brit. J. Gen. Prac..

[35] Park H.Y., Seo S.A., Yoo H., Lee K. (2018). Medication adherence and beliefs about medication in elderly patients living alone with chronic diseases. Patient Prefer. Adherence.

[36] Crocker T.F., Brown L., Clegg A., Farley K., Franklin M., Simpkins S., Young J. (2019). Quality of life is substantially worse for community-dwelling older people living with frailty: Systematic review and meta-analysis. Qual. Life Res..

[37] Rausch C., Liang Y., Bültmann U., De Rooij S.E., Johnell K., Laflamme L., Möller J. (2019). Social position and geriatric syndromes among Swedish older people: A population-based study. BMC Geriatr..

[38] Lee J.K. (2013). Evaluation of a medication self-management education program for elders with hypertension living in the community. J. Korean Acad. Nurs..

[39] Min Y.M., Sull J.W., Ohrr H.C., Lee E.S. (2009). A study on the awareness and behavior of consumers and pharmacists toward non prescription drugs. J. Pharmacoepidemiol. Risk Manage..

[40] Geriatrics Society Beers Criteria Update Expert Panel (2019). American geriatrics society updated beers criteria for potentially inappropriate medication use in older adults. J. Am. Geriatr. Soc..

[41] Beers M.H., Ouslander J.G., Rollingher I., Reuben D.B., Brooks J., Beck J.C. (1991). Explicit criteria for determining inappropriate medication use in nursing home residents. Arch. Intern. Med..

[42] Rochon P., Schmader K., Sokol H. (2014). Drug Prescribing for Older Adults. https://www.uptodate.com/contents/drug-prescribing-for-older-adults.

[43] Suh Y., Ah Y.-M., Han E., Jun K., Hwang S., Choi K.H., Lee J.-Y. (2020). Dose response relationship of cumulative anticholinergic exposure with incident dementia: Validation study of Korean anticholinergic burden scale. BMC Geriatr..

[44] Hope C.J., Wu J., Tu W., Young J., Murray M.D. (2004). Association of medication adherence, knowledge, and skills with emergency department visits by adults 50 years or older with congestive heart failure. Am. J. Health Pharm..

[45] O’Connell M.B., Johnson J.F. (1992). Evaluation of medication knowledge in elderly patients. Ann. Pharmacother..

[46] Barat I., Andreasen F., Damsgaard E.M.S. (2001). Drug therapy in the elderly: What doctors believe and patients actually do. Br. J. Clin. Pharmacol..

[47] Tesfamariam S., Anand I.S., Kaleab G., Berhane S., Woldai B., Habte E., Russom M. (2019). Self-medication with over the counter drugs, prevalence of risky practice and its associated factors in pharmacy outlets of Asmara, Eritrea. BMC Public Health.

[48] Kim Y.-S., Kim H.S., Kim H.A., Chun J., Kwak M.J., Kim M.-S., Hwang J.-I., Kim H. (2020). Can patient and family education prevent medical errors? A descriptive study. BMC Health Serv. Res..

[49] Wirawan A.A., Hutajulu S.H., Haryani H. (2020). The effect of prechemotherapy education using audio visual methods on the distress of patients with cancer. J. Cancer Educ..

[50] Jyotsna V.P., Rahaman H.S., Sreenivas V., Krishnan A., Tandon N. (2018). Effectiveness of a patient education module on diabetic foot care in outpatient setting: An open-label randomized controlled study. Indian J. Endocrinol. Metab..

[51] Neumann-Podczaska A., Seostianin M., Madejczyk K., Merks P., Religioni U., Tomczak Z., Tobis S., Moga D., Ryan M., Wieczorowska-Tobis K. (2021). An experimental education project for consultations of older adults during the pandemic and healthcare lockdown. Healthcare.

